# Trends in Alcohol Intake and the Association between Socio-Demographic Factors and Volume of Alcohol Intake amongst Adult Male Drinkers in China

**DOI:** 10.3390/ijerph16040573

**Published:** 2019-02-16

**Authors:** Ruiyi Liu, Li Chen, Fan Zhang, Rui Zhu, Xinjie Lin, Xuchen Meng, Huabing Li, Xun Lei, Yong Zhao

**Affiliations:** 1 School of Public Health and Management, Chongqing Medical University, Chongqing 400016, China; lry981118@foxmail.com (R.L.); nameclx@foxmail.com (L.C.); ava11@126.com (F.Z.); 2016111041@stu.cqmu.edu.cn (R.Z.); lhbcqmu@outlook.com (H.L.); 2 Research Center for Medicine and Social Development, Chongqing Medical University, Chongqing 400016, China; 3 The Innovation Center for Social Risk Governance in Health, Chongqing Medical University, Chongqing 400016, China; 4 Clinical College, Chongqing Medical University, Chongqing 400016, China; linxinjie1@outlook.com (X.L.); angelitamxc@hotmail.com (X.M.); 5 Chongqing Key Laboratory of Child Nutrition and Health. Chongqing 400016, China

**Keywords:** volume, alcohol type, alcohol intake, age, China, socio-demographic

## Abstract

*Background*: The volume of alcohol intake and type of alcohol affect Chinese men’s health. This study investigated changes of alcohol type between 2004 and 2011, explored the trend of change in alcohol type with age and determined the social demographic factors influencing the alcohol intake of Chinese men. *Methods*: Research data originated from the public database, China Health and Nutrition Survey (CHNS). Three chi-square tests were used to determine the prevalence of different alcohol types (beer, wine and liqueur) and the trend with age among male drinkers from 2004 to 2011. An ordered logistic regression model was established with alcohol intake as the dependent variable and social demography as the independent variable to analyze the influence of these factors on male alcohol intake. *Results*: This study confirmed that from 2004 to 2011, 70.1% of Chinese men consumed alcohol less than 168 g/w. The popularity of beer was on the rise, while the liqueur alcohol consumption decreased from 2004 to 2011 and the consumption of wine began to rise rapidly after 2006 (*p* < 0.05 for all). The prevalence of liqueur drinking increased with age and the prevalence of beer drinking decreased with age among Chinese male drinkers (*p* < 0.05 for all). From 2004 to 2011, a positive correlation appeared between age and male alcohol intake (*p* < 0.05 for all). In 2004 (OR = 1.22, 95% CI: 1.03–1.44), 2006 (OR = 1.21, 95% CI: 1.02–1.42) and 2011 (OR = 1.51, 95% CI: 1.31–1.75), Chinese men living in rural areas had a high volume of alcohol intake. From 2004 to 2011, the participants had married consumed more alcohol (*p* < 0.05 for all). In 2004 (OR = 0.61, 95% CI: 0.43–0.88) and 2011 (OR = 0.80, 95% CI: 0.68–0.94), higher education levels were negatively correlated with male alcohol intake. In 2006 (OR = 1.29, 95% CI: 1.07–1.56), 2009 (OR = 1.76, 95% CI: 1.45–2.14) and 2011 (OR = 1.35, 95% CI: 1.13–1.61), male drinkers who were working consumed more alcohol. From 2004 to 2011, a significant positive correlation appeared between tobacco consumption and alcohol intake (*p* < 0.05 for all). *Conclusion*: Consumption of three types of alcohol (beer, wine and liqueur) varies with the year. Beer consumption decreases with age, whereas liqueur consumption increases with age. Social demographic factors, such as residence, age, highest education level, working status and tobacco consumption, are related to alcohol intake. Our study affirms the effect of age on the choice of different types of alcohol.

## 1. Introduction

The volume of alcohol intake is an important factor in determining the effect of alcohol on chronic and acute health outcomes in people [1]. Since 2000, the percentage of global drinkers has fallen from 47.6% to 43.0%, indicating a decline of nearly 5% points [1]. However, in the Western Pacific region dominated by China, the proportion of current drinkers is rising. In 2012, China had 54.6% male drinkers [2]. The world’s total per capita alcohol consumption rose from 5.5 liters in 2005 to 6.4 liters in 2010. Meanwhile, the per capita alcohol consumption in China rose from 4.1 liters in 2005 to 7.1 liters in 2010, higher than the world average. In 2016, the total per capita alcohol consumption of Chinese men reached 17.0 liters [1]. Liqueur and beer are the most common types of alcohol consumed by Chinese drinkers. In 2016, liqueur accounted for 67% and beer for 30%. Large numbers of Chinese male drinkers, whose high per capita consumption and preference for liqueur, increased the risk of alcohol use disorders [3].

The adverse effects of alcohol consumption are widely studied worldwide. Diseases, such as coronary heart disease [4,5], liver cirrhosis [6] and ischemic stroke [7,8], are closely related to heavy alcohol intake. A 2010 study reported that moderate to heavy drinking increased the risk of coronary heart disease among Chinese men [9]. Over the past 30 years, because of heavy alcohol consumption, the number of patients with alcoholic liver disease (ALD) in China has been increasing at an alarming rate, with 98% of them being male [10]. Heavy drinking significantly increases the risk of ischemic stroke in Chinese men [11]. Any level of alcohol consumption is a risk factor for cancer [12,13]. Alcohol consumption increases the risk of stomach and liver cancer among Chinese men [14]. Nonetheless, alcohol is a particularly important way to socialize in China [15]. Liqueur is the most popular type of alcohol and most Chinese men choose to drink it. However, studies have affirmed that the consumption of liqueur is closely related to the incidence of esophageal cancer [16].

Alcohol intake and type affect the physical health of Chinese male drinkers. Many studies have contended that alcohol consumption of Chinese men is influenced by social demographic factors. Residence, age, highest education level, working status and tobacco consumption all affect alcohol consumption. Rural Chinese men drink more alcohol than urban men because of their homemade alcoholic drinks [17]. Older Chinese men drink more alcohol than younger men. Men with high levels of education had lower daily alcohol intake and working men were more likely to drink than non-working men [18,19]. Tobacco and alcohol have a strong correlation and the increase of tobacco consumption will increase alcohol consumption but more research is needed in China to clarify this point [20].

In recent years, the choice of Chinese male drinkers has been dominated by liqueur, followed by beer and wine [19]. Liqueur and beer consumption has been rising since 2004 [21,22]. With the growth of Chinese people’s income and the upgrading of the consumption concept, the consumption of wine began to accelerate [21]. Meanwhile, older Chinese men are more likely to consume liqueur, while 18 to 35-year-old Chinese men are more likely to consume wine [21,23].

Although many studies have described extensively the current drinking behavior of Chinese men, few have explored the change in male drinking behavior and the reasons behind it. Our study further confirms these changes and sheds light on possible causes. Current research confirms that different age groups have different preferences for alcohol types, which suggests different interventions can be implemented for different age groups. We propose that socio-demographic factors influence male drinking patterns in China and these factors provide information about adverse lifestyle and health outcomes caused by drinking. This information is critical for assessing the extent and trends of alcohol-related hazards, enhancing advocacy and assessing the impact of existing interventions. The present study used data from the China Health and Nutrition Survey (CHNS) between 2004 and 2011 to investigate the drinking status of Chinese men. Our research objectives are to explore the (1) trend of the selection of the different types of alcohol with the change of year, (2) trend of selecting the different types of alcohol with age group and (3) the association between alcohol consumption of Chinese males and socio-demographic variables from 2004 to 2011. The findings of the present study could assist evidence-based policy-making regarding alcohol use among adult males in China.

## 2. Materials and Methods 

### 2.1. Study Design: CHNS

The CHNS, taking nine provinces (Liaoning, Jiangsu, Shandong, Henan, Hubei, Hunan, Guangxi, Guizhou and Heilongjiang) and three large cities (Beijing, Shanghai and Chongqing, which were added in 2011) as study sites, aims to study China’s health, population, socio-economic and nutrition policies [24]. All respondents were asked if they drank alcohol, such as liqueur, beer, wine or any other alcoholic beverage. The study included 10,648 participants who reported their alcohol consumption and at least one defined alcohol intake. Among the total participants, 2397 were in 2004; 2375 in 2006; 2588 in 2009 and 3288 in 2011. We included all adults who reported their drinking while excluding data with missing values. At least one alcohol intake was reported in all included samples. The present study performed analyses with the CHNS data published at the official website [25].

### 2.2. Alcohol Consumption

The outcome variable was the volume of alcohol intake for a week, which included three types of alcohol intake. The three alcohol types are beer, wine and liqueur. If the participants had used some type of alcohol, they were asked the following three questions: (1) Do you drink beer? (2) Do you drink grape wine (including various colored wines and rice wine)? and (3) Do you drink liqueur? The participants were asked three questions about how much of each alcohol they drank: (1) How much beer (bottle) do you drink each week? (2) How much grape wine (including various colored and rice wine) do you drink each week (Liang)? and (3) How much liqueur do you drink each week (Liang)? The alcohol concentration of different alcoholic beverages was in accordance with the 2010 China monitoring report on chronic disease risk factors (beer = 4%, grape wine = 10% and liqueur = 38%).

1 bottle = 330 ml, 1 Liang = 50 ml. Alcohol intake = average per drink (ml) * alcohol concentration 0.8

In the following, we provide a calculation method for the volume of alcohol contained in three different drinks and a calculation method for the total volume of the alcohol consumed.

A: Alcohol intake (beer) = bottle * 330 ml * 0.04.

B: Alcohol intake (grape wine) = Liang * 50 ml * 0.1.

C: Alcohol intake (liqueur) = Liang * 50 ml * 0.38.

Total alcohol intake = A + B + C.

According to the final calculation results and previous studies [26], the alcohol consumption of Chinese men was divided into four levels: ≤ 84 g/w, 84.01–168 g/w, 168.01–336 g/w and ≥ 336.01 g/w.

### 2.3. Socio-Demographic Variables

Socio-demographic variables including residence, age, marital status, highest education levels, working status and tobacco consumption were used in the analyses. The participants were divided into groups, namely, for residence, into urban and rural; for marital status, into spinsterhood, married and other (divorce, widowed, separated and unknown); for educational attainment, into unfinished primary school and below, secondary and primary school graduates and university or above; for working status, respondents who are working and are not and for tobacco consumption, into smoker and non-smoker.

### 2.4. Data Analyses

Frequencies and percentages were used to assess the proportion of categorical variables. The means and standard error were used to describe continuous variables. Three chi-square tests were performed to establish the trend and to determine the prevalence of different types of alcohol consumption among male drinkers from 2004 to 2011 and the trend in the prevalence of alcohol consumption (beer, wine and liqueur) among male drinkers with age. The ordered logistic regression model was conducted with the volume of alcohol intake as dependent variable and with residence (urban and rural), age, marital status (spinsterhood, married and other), highest education levels (unfinished primary school and below, secondary and primary school graduates and university or above), working status (yes and no) and tobacco consumption (yes and no) as independent variables. All tests were two-sided and p-values less than 0.05 were considered statistically significant. All data analyses were performed using statistical software SPSS version 22.0 (SPSS Inc., Chicago, IL, USA).

## 3. Results

### 3.1. Characteristics of the Sample

A total of 10,648 adult male participants from the CHNS data were included in this study. All participants were drinkers and 71.5% of them smoked. The average age of all the participants was 48.22 ± 13.74. Around 36.3% of the participants lived in cities, while 63.7% lived in rural areas. A total of 88.5% of the participants were married. Lastly, 67.7% of the participants had attended unfinished primary school and below, 23.7% had attended junior and senior high schools and only 8.6% had a college degree or above (Table 1).

### 3.2. Volume of Alcohol Intake in Male Drinkers

The current study investigated adult male alcohol consumption between 2004 and 2011. Of the 10,648 participants, 50.1% consumed no more than 84 g/w of alcohol, 20.0% consumed 84.01–168 g/w of alcohol, 18.0% drank 168.01–336 g/w and 11.9% drank no less than 336.01 g/w (Table 2).

### 3.3. Trends in the Prevalence of the Different Types of Alcohol Consumption by Survey Year

**Figure 1:**
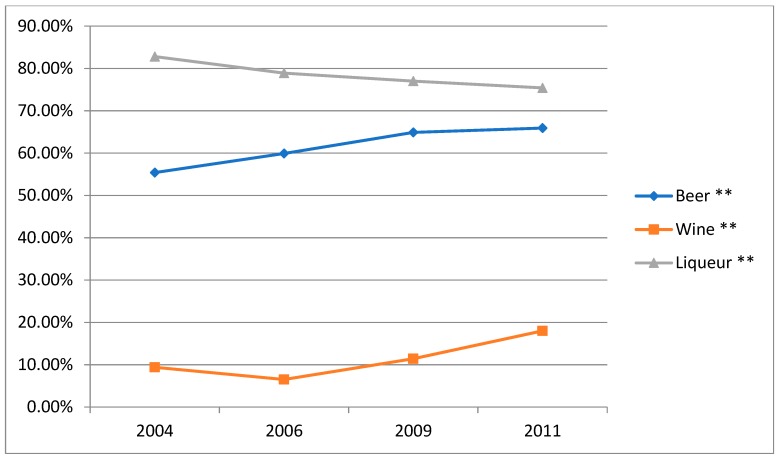
Trends in the prevalence of the different types of alcohol consumption amongst male drinkers by survey year, CHNS. ** Statistically significant (*p* < 0.01).

The three types of alcohol consumption changed significantly with the survey years. From 2004 to 2011, the beer alcohol consumption increased significantly, while the liqueur alcohol consumption decreased. From 2006 to 2011, the wine alcohol consumption increased significantly (Figure 1). The detailed data are shown in Table S1 of the supplementary material.

### 3.4. Time Trends in the Prevalence of Alcohol Consumption (Beer, Wine and Liqueur) amongst Male Drinkers by Age Groups

**Figure 2:**
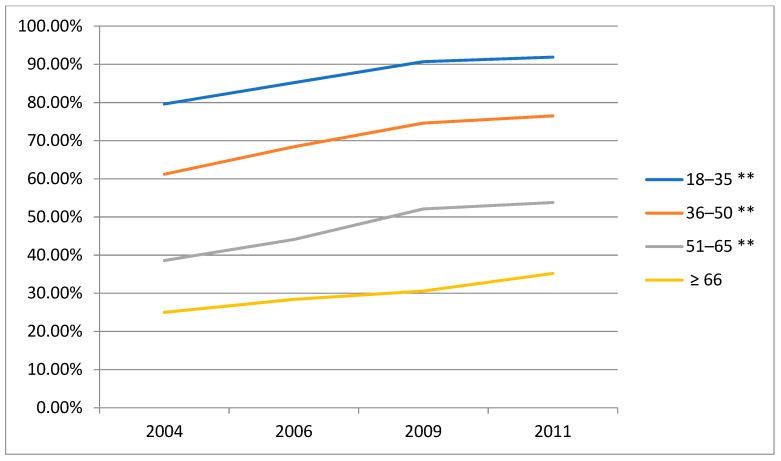
Time trends in the prevalence of alcohol consumption (beer) amongst male drinkers by age groups, CHNS. ** Statistically significant (*p* < 0.01).

**Figure 3:**
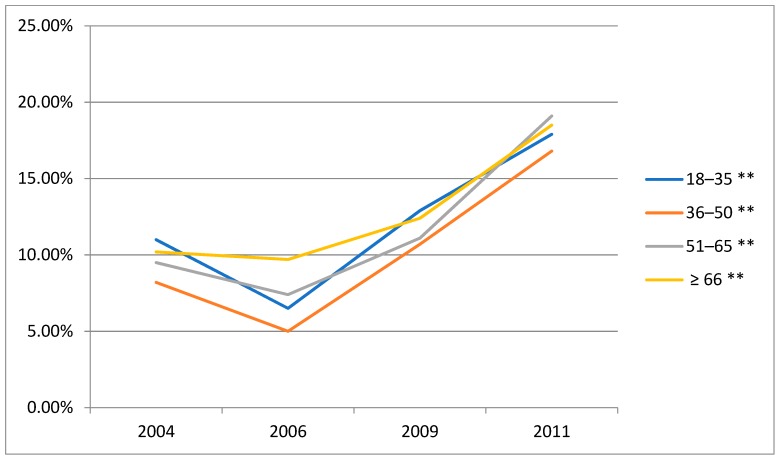
Time trends in the prevalence of alcohol consumption (wine) amongst male drinkers by age groups, CHNS. ** Statistically significant (*p* < 0.01).

**Figure 4:**
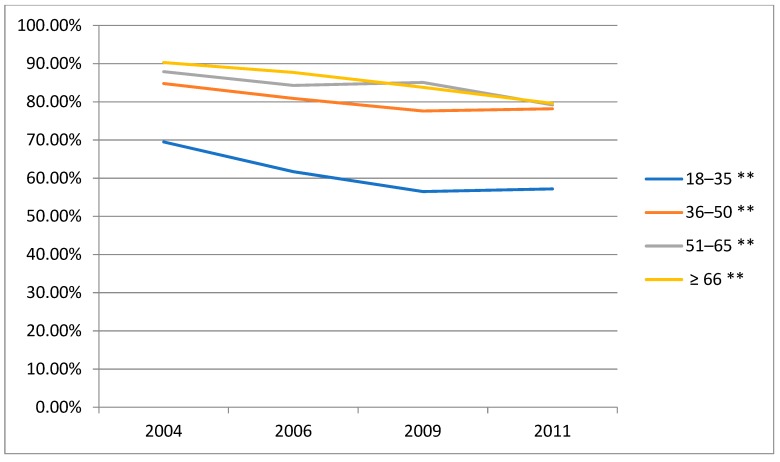
Time trends in the prevalence of alcohol consumption (liqueur) amongst male drinkers by age groups, CHNS. ** Statistically significant (*p* < 0.01).

The beer alcohol consumption increased significantly from 2004 to 2011 (Figure 2). Wine alcohol intake decreased from 2004 to 2006, while increased from 2006 to 2011 (Figure 3). The liqueur alcohol consumption decreased slightly from 2004 to 2011 (Figure 4). The detailed data can be obtained from Table S2 of the supplementary material. What’s more, according to these figures, the beer alcohol consumption decreased significantly with age in 2004, 2006, 2009 and 2011. Wine alcohol intake changed irregularly with age from 2004 to 2011. The liqueur alcohol consumption increased significantly with age in 2004, 2006, 2009 and 2011.

### 3.5. Ordered Logistic Regression Model Analysis for the Volume of Alcohol Intake amongst Chinese Male Drinkers

Our data confirmed the findings that age from 2004 to 2011 was positively correlated with alcohol intake. Males living in rural areas tended to consume more alcohol in 2004 (OR = 1.22, 95% CI: 1.03–1.44), 2006(OR = 1.21, 95% CI: 1.02–1.42) and 2011(OR = 1.51, 95% CI: 1.31–1.75). From 2004 to 2011, the participants had married consumed more alcohol. In 2004(OR = 0.61, 95% CI: 0.43–0.88) and 2011(OR = 0.80, 95% CI: 0.68–0.94), the participants had higher education level consumed less alcohol. In 2006(OR = 1.29, 95% CI: 1.07–1.56), 2009 (OR = 1.76, 95% CI: 1.45–2.14) and 2011(OR = 1.35, 95% CI: 1.13–1.61), a significant positive correlation appeared between working status and volume of alcohol intake. From 2004 to 2011, the volume of alcohol intake increased with tobacco consumption (Table 3).

## 4. Discussion

The current study reported the volume of alcohol consumed by male drinkers in China from 2004 to 2011 and identified the factors that affected the drinking. From 2004 to 2011, beer consumption in the Chinese male population increased significantly, while the liqueur alcohol consumption decreased. From 2006 to 2011, the number of Chinese men consuming wine increased significantly. Men of different ages tended to consume different types of alcohol. Social demographic factors, such as age, residence, education levels, working status and smoking status, were associated significantly with the volume of alcohol intake.

Our study validated most Chinese men consume light to moderate volumes of alcohol and a few men are heavy consumers. Light and moderate alcohol intake is affirmed to be good for certain diseases but that benefit is offset when considering the overall health risks, such as cancer and accidental injury [27]. China has seen a marked increase in alcohol-related public health problems in recent years and even light and moderate drinking cannot ignore its significant health effects [17,28,29]. From 2004 to 2011, Chinese men’s beer consumption increased significantly while liqueur consumption decreased. After 2006, wine became popular among Chinese men. Drinkers think wine is healthier than liqueur because of lower alcohol content. Moreover, wine represents high taste and fashion, making young men more inclined to consume wine [30].

The consumption of beer and liqueur showed different trends with age. From 2004 to 2011, beer was much preferred among young people over the other two alcoholic drinks. Eighteen to thirty-five-year-old males were most likely to drink beer. Beer is relatively inexpensive and easy to obtain and popular among Chinese students. However, the under-18 population is exposed to beer and can easily drink it continuously [31,32]. Drinking beer may be a sign of a shift towards smoking and heavy intake of alcohol and other drugs [33]. Although alcohol intake by minors is frowned upon by law and society, the availability of alcohol to minors is very high [34]. Hence, alcohol-related health education is necessary for minors. Community-based interventions can effectively reduce high-risk alcohol consumption and effects of alcohol [35,36], especially for the youth. Liqueur consumption trends show the opposite from that of beer consumption, as more Chinese men are more inclined to consume beer as they age. In China, older men are likely to use liqueur in social situations to liven up the atmosphere or increase feelings [37]. Additionally, the WHO estimates that 25% of the alcohol consumed in China is unregistered, including large volumes of home-made liqueur and liqueur produced by unregulated distilleries [38,39,40]. The older the men, the more likely they are to consume unrecorded liqueur [38]. Notably, despite WHO’s recommendations to reduce alcohol advertising to control people’s alcohol intake, China still has many ways of advertising alcohol, including liqueur and beer. Advertisers have misled consumers into thinking that liqueur symbolizes freedom, maturity and good relationships. 

Participants who lived in rural areas drank more alcohol than participants who lived in urban areas in 2004, 2006 and 2011. A large sample of previous studies in China validated that drinking rates in rural areas were higher than in urban areas [41]. Furthermore, the prevalence rate of three types of alcohol in rural areas was higher than that in urban areas [41]. Meanwhile, the consumption of liqueur was more common in rural China. Associated with traditional culture, a considerable volume of homemade alcohol had appeared in rural China [17]. This scenario increases alcohol consumption among men in rural areas. In another study of Chinese men, 165,000 (more than three-quarters) were drinkers from rural areas [42]. This scenario claims the problem of alcohol consumption in rural areas deserves more attention from the Chinese government and active control measures are needed to intervene in alcohol consumption.

Older participants drank more alcohol than younger ones from 2004 to 2011. A study conducted in two major provinces of China asserted the drinking rate increased with age and that the drinking rate was higher in the higher age group [17]. Death rates from alcohol-related chronic diseases increased with age [43]. Numerous studies have affirmed a significant relationship between alcohol-related chronic diseases and age. Older drinkers are drinking more alcohol, perhaps suggesting that health workers should pay more attention to older drinkers to prevent more serious consequences.

The current study also found that married Chinese men drank more alcohol from 2004 to 2011. Chinese men play a very important role in the family and they are under greater pressure to make their families have a higher quality of life. Previous studies have found that a good marriage reduces men’s alcohol consumption by eliminating unnecessary social situations [44]. Therefore, maintaining good family relationships is necessary to reduce the amount of alcohol Chinese men drink.

Participants with higher education levels consumed less alcohol than participants with lower education levels in 2004 and 2011. Education levels affect attitudes toward alcohol consumption, which affect behavior and may explain the findings of this study. Male drinkers who were working drank more alcohol than drinkers who were not working in 2006, 2009 and 2011. In China, men drink more alcohol for work reasons. Drinking is considered a good way to socialize in China, especially among men. Furthermore, people believe that moderate drinking can reduce the high pressure caused by work and relieve the body and mind. Work status may further affect income level. Studies have shown that people at higher income levels were more likely to drink and consume more alcohol [45,46].

Male drinkers who smoked drank more alcohol than participants who did not from 2004 to 2011. A previous study affirmed that tobacco consumption was significantly associated with alcohol consumption [47]. Alcohol and tobacco are addictive substances, which interact with each other, with one addictive substance increasing the use of the other [20]. Alcohol and tobacco are social objects in China. Socially, alcohol and tobacco serve as catalysts and many men believe that tobacco and alcohol can bring people closer together quickly. Hence, when a man uses tobacco, other men use words to encourage him to drink more alcohol. Men are more likely to receive encouragement from peers to drink more alcohol.

Worldwide, 44.8% of the total recorded alcohol is consumed in the form of liqueur. The second most consumed type of beverage is beer (34.3%) and is followed by wine (11.7%). This type of alcohol preference has changed only slightly since 2010. The main change has been in Europe, where the recorded consumption of liqueur has fallen while the ratio of wine and beer has risen. In other parts of the world, such as Japan, no significant change was observed in the consumption trend of alcohol but a significant upward trend was seen in China. At the same time, alcohol is causing serious health problems for Chinese men and therefore, effective alcohol and public health surveillance systems need to be established. The system should cover three main areas, namely, alcohol consumption, the health consequences of alcohol use and alcohol policy and program responses and corresponding impact. An investigation is needed on the social and demographic factors related to alcohol consumption for policy making and the establishment of the detection system. The advantages of this study are reflected in the data adopted in this study are based on national survey data, which are representative. And this study provides practical evidence for different age groups in choosing the type of alcohol.

This study also has limitations. First, as in most observational studies, all data were obtained through self-reports and inaccurate recall or under-reporting may affect the results. Future studies could consider using more accurate measurement tools to obtain the accurate alcohol intake of participants over time. Second, the socio-demographic factors contained in this study are incomprehensive. Third, social and environmental factors were not included in this study. However, social and environmental factors influence alcohol intake. Future research can consider design to include more possible influencing factors. Finally, the proportions (or sample size) of several variables’ stratum (e.g., marital status) are observed as being small, which may result in less precision for the model due to having a large variance. 

## 5. Conclusions

From 2004 to 2011, Chinese men’s beer consumption increased steadily while their consumption of liqueur decreased. Since 2006, more men have chosen to consume wine. Chinese men of different ages chose different types of alcohol. Young men tended to consume beer, while older men preferred liqueur. Chinese men’s alcohol consumption between 2004 and 2011 was influenced by socio-demographic factors, such as residence, age, highest education level, working status and tobacco consumption. Our findings suggest that Chinese men of different ages should be provided with different interventions to address the differences in the type and amount of alcohol they consume. In the future, the socio-demographic factors affecting male alcohol patterns in different age groups can be further explored to promote the development of health strategies.

## Figures and Tables

**Table 1:** Characteristics of 10,648 participants stratified by the survey year (*n*, %), CHNS.

Variable		2004	2006	2009	2011	Total
Residence	Urban	837 (34.9%)	810 (34.1%)	895 (34.6%)	1321 (40.2%)	3863 (36.3%)
	Rural	1560 (65.1%)	1565 (65.9%)	1693 (65.4%)	1967 (59.8%)	6785 (63.7%)
Age		46.70 ± 13.71	47.75 ± 13.54	48.33 ± 13.92	35.34 ± 8.09	48.22 ± 13.74
Marital status	Spinsterhood	201 (8.4%)	157 (6.6%)	180 (7.0%)	172 (5.2%)	710 (6.7%)
	Married	2090 (87.2%)	2110 (88.8%)	2285 (88.3%)	2939 (89.4%)	9424 (88.5%)
	Other ^1^	106 (4.4%)	108 (4.5%)	123 (4.8%)	177 (5.4%)	514 (4.8%)
Highest education levels	PSB ^2^	1709 (71.3%)	1606 (67.7%)	1817 (70.2%)	2081 (63.3%)	7213 (67.7%)
	SPSG ^3^	557 (23.2%)	602 (25.3%)	591 (22.8%)	769 (23.4%)	2519 (23.7%)
	UA ^4^	131 (5.5%)	167 (7.0%)	180 (7.0%)	438 (13.3%)	916 (8.6%)
Working status	Yes	1769 (73.8%)	1748 (73.6%)	1921 (74.2%)	2422 (73.7%)	7860 (73.8%)
	No	628 (26.2%)	627 (26.4%)	667 (25.8%)	866 (26.3%)	2788 (26.1%)
Tobacco consumption	Yes	1750 (73.0%)	1718 (72.3%)	1807 (69.8%)	2342 (71.2%)	7617 (71.5%)
	No	647 (27.0%)	657 (27.7%)	781 (30.2%)	946 (28.8%)	3031 (28.5%)

^1^ Divorce, widowed, separated and unknown; ^2^ Unfinished primary school and below; ^3^ Secondary and primary school graduates; ^4^ University or above.

**Table 2:** Distribution of 10,684 males with different alcohol intakes during the survey year (*n*, %), CHNS.

Volume of Alcohol Intake	2004	2006	2009	2011	Total
≤84 g/w	1065 (44.4%)	1087 (45.8%)	1375 (53.1%)	1811 (55.1%)	5338 (50.1%)
84.01–168 g/w	469 (19.6%)	470 (19.8%)	515 (19.9%)	679 (20.7%)	2133 (20.0%)
168.01–336g/w	502 (20.9%)	464 (19.5%)	429 (16.6%)	518 (15.8%)	1913 (18.0%)
≥336.01 g/w	361 (15.1%)	354 (14.9%)	269 (10.4%)	280 (8.5%)	1264 (11.9%)
Mean ± SD	181.25 ± 209.77	181.53 ± 224.59	142.48 ± 181.79	129.70 ± 161.27	155.97 ± 194.31

Notes: g/w means gram/week.

**Table 3:** Ordered logistic regression model analysis for the volume of alcohol intake amongst Chinese male drinkers, CHNS.

Volume of Alcohol Intake ^1^					
Survey Year		2004	2006	2009	2011
Parameter		OR (95% CI)	OR (95% CI)	OR (95% CI)	OR (95% CI)
Intercept1		5.56 (3.60–8.57)	5.98 (3.75–9.53)	13.41 (8.38–21.46)	9.44 (5.91–15.07)
Intercept2		12.77 (8.22–19.81)	13.85 (8.65–22.20)	33.45 (20.74–54.00)	24.88 (15.49–39.96)
Intercept3		42.18 (26.84–66.29)	42.78 (26.39–69.27)	110.61 (67.56–180.91)	88.15 (54.16–143.31)
Age	—	1.02 (1.02–2.06)	1.02 (1.01–2.05)	1.02 (1.02–2.06)	1.02 (1.01–2.04)
Residence	Rural	1.22 (1.03–1.44)	1.21 (1.02–1.42)	1.13 (0.96–1.33)	1.51 (1.31–1.75)
	Urban (ref)	1.00	1.00	1.00	1.00
Marital status	Other	1.35 (0.85–2.16)	1.35 (0.80–2.27)	1.18 (0.70–1.99)	2.02 (1.26–3.25)
	Married	1.46 (1.08–1.98)	1.80 (1.25–2.59)	1.68 (1.15–2.45)	1.71 (1.17–2.50)
	Single (ref)	1.00	1.00	1.00	1.00
Highest education levels	UA ^4^	0.61 (0.43–0.88)	0.88 (0.64–1.19)	1.17 (0.86–1.58)	0.99 (0.80–1.23)
	SPSG ^3^	1.04 (0.87–1.25)	0.96 (0.80–1.15)	0.86 (0.71–1.03)	0.80 (0.68–0.94)
	PSB ^2^ (ref)	1.00	1.00	1.00	1.00
Working status	Yes	1.14 (0.94–1.37)	1.29 (1.07–1.56)	1.76 (1.45–2.14)	1.35 (1.13–1.61)
	No (ref)	1.00	1.00	1.00	1.00
Tobacco consumption	Yes	1.73 (1.45–2.05)	1.59 (1.34–1.89)	1.85 (1.56–2.19)	1.63 (1.40–1.90)
	No (ref)	1.00	1.00	1.00	1.00

^1^ Four level variables: ≤ 84 g/w, 84.01–168 g/w, 168.01–336 g/w, ≥ 336.01 g/w. ^2^ Unfinished primary school and below; ^3^ Secondary and primary school graduates; ^4^ University or above.

## Supplementary Materials

The following are available online at http://www.mdpi.com/1660-4601/16/4/573/s1, Table S1: Trends in prevalence of different types of alcohol consumption among male drinkers by survey year, Table S2: Time trends in the prevalence of alcohol consumption (beer, wine, liqueur) amongst male drinkers by age groups, CHNS.
