# Supplementary material for: Trends in Alcohol Intake and the Association between Socio-Demographic Factors and Volume of Alcohol Intake amongst Adult Male Drinkers in China
Source: Int J Environ Res Public Health. 2019 Feb 16;16(4):573. doi: 10.3390/ijerph16040573 (PMC6406336; doi:10.3390/ijerph16040573)
Supplement: Supplementary file 1 [file ijerph-16-00573-s001.pdf]

International Journal of
Environmental Research and Public Health

MDPI

Supplementary Material

Trends in Alcohol Intake and the Association between Socio-Demographic Factors and Volume of Alcohol Intake amongst Adult Male Drinkers in China

Ruiyi Liu [1,2,3,†], Li Chen [1,2,3,†], Fan Zhang [1,2,3], Rui Zhu [1,2,3], Xinjie Lin [4], Xuchen Meng [4], Huabing Li [1,2,3], Xun Lei [1,2,3,*] and Yong Zhao [1,2,3,5*]

[1] School of Public Health and Management, Chongqing Medical University, Chongqing 400016, China; lry981118@foxmail.com (R.L.); nameclx@foxmail.com (L.C.); ava11@126.com (F.Z.); 2016111041@stu.cqmu.edu.cn (R.Z.); lhbcqmu@outlook.com (H.L.)
[2] Research Center for Medicine and Social Development, Chongqing Medical University, Chongqing 400016, China
[3] The Innovation Center for Social Risk Governance in Health, Chongqing Medical University, Chongqing 400016, China
[4] Clinical College, Chongqing Medical University, Chongqing 400016, China; linxinjie1@outlook.com (X.L.); angelitamxc@hotmail.com (X.M.)
[5] Chongqing Key Laboratory of Child Nutrition and Health. Chongqing 400016, China;
† These authors contributed equally to this work.
* Correspondence: zhaoyong@cqmu.edu.cn (Y.Z.); leixun521@163.com (X.L.); Tel.: +86-23-6848-5008 (Y.Z.); +86-23-6848-5031 (X.L.)

Table S1. Trends in prevalence of different types of alcohol consumption among male drinkers by survey year.

Variable			2004	2006	2009	2011	Pearson Chi-Square	p-Value
Beer consumption	Non-beer-eater	N	1070	952	908	1121	79.81	0.000
		%	44.6	40.1	35.1	34.1		
	Beer-eater	N	1327	1423	1680	2167		
		%	55.4	59.9	64.9	65.9		
Wine consumption	Non-wine-eater	N	2172	2220	2292	2695	198.18	0.000
		%	90.6	93.5	88.6	82.0		
	Wine-eater	N	225	155	296	593		
		%	9.4	6.5	11.4	18.0		
Liqueur consumption	Non-liqueur-eater	N	413	501	594	809	47.30	0.000
		%	17.2	21.1	23.0	24.6		
	Liqueur-eater	N	1984	1874	1994	2479		
		%	82.8	78.9	77.0	75.4		

Table S2. Time trends in the prevalence of alcohol consumption (beer, wine, liqueur) amongst male drinkers by age groups, CHNS.

			Beer				Wine				Liqueur			
**Age groups**			**2004**	**2006**	**2009**	**2011**	**2004**	**2006**	**2009**	**2011**	**2004**	**2006**	**2009**	**2011**
18-35	Non drinker	N	111	69	43	42	485	435	404	428	166	178	202	223
		%	20.4	14.8	9.3	8.1	89.0	93.5	87.1	82.1	30.5	38.3	43.5	42.8
	drinker	N	434	396	421	479	60	30	60	93	379	287	262	298
		%	79.6	85.2	90.7	91.9	11.0	6.5	12.9	17.9	69.5	61.7	56.5	57.2
36-50	Non drinker	N	360	289	244	278	853	868	856	984	141	175	215	258
		%	38.8	31.6	25.4	23.5	91.8	95.0	89.3	83.2	15.2	19.1	22.4	21.8
	drinker	N	569	625	715	905	76	46	103	199	788	739	744	925
		%	61.2	68.4	74.6	76.5	8.2	5.0	10.7	16.8	84.8	80.9	77.6	78.2
51-65	Non drinker	N	422	425	419	560	622	704	777	980	83	119	130	252
		%	61.4	55.9	47.9	46.2	90.5	92.6	88.9	80.9	12.1	15.7	14.9	20.8
	drinker	N	265	335	455	652	65	56	97	232	604	641	744	960
		%	38.6	44.1	52.1	53.8	9.5	7.4	11.1	19.1	87.9	84.3	85.1	79.2
≥66	Non drinker	N	177	169	202	241	212	213	255	303	23	29	47	76
		%	75.0	71.6	69.4	64.8	89.8	90.3	87.6	81.5	9.7	12.3	16.2	20.4
	drinker	N	59	67	89	131	24	23	36	69	213	207	244	296
		%	25.0	28.4	30.6	35.2	10.2	9.7	12.4	18.5	90.3	87.7	83.8	79.6

© 2019 by the authors. Submitted for possible open access publication under the terms and conditions of the Creative Commons Attribution (CC BY) license (http://creativecommons.org/licenses/by/4.0/).
